# Evaluation of the Course of Inferior Alveolar Canal and its Relation to Anatomical Factors on Digital Panoramic Radiographs

**DOI:** 10.30476/DENTJODS.2020.87973.1304

**Published:** 2021-09

**Authors:** Ali Derafshi, Khalil Sarikhani, Farzaneh Mirhosseini, Motahareh Baghestani, Roghaye Noorbala, Motahareh Kaboodsaz Yazdi

**Affiliations:** 1 Postgraduated Student, Dept. of Oral and Maxillofacial Radiology, School of Dentistry, Shahid Sadoughi University of Medical Sciences, Yazd, Iran; 2 Oral and Maxillofacial Radiologist, Shiraz, Iran; 3 Oral and Maxillofacial Radiologist, Yazd, Iran; 4 Dept. of Oral and Maxillofacial Radiology, School of Dentistry, Shahid Sadoughi University of Medical Sciences, Yazd, Iran; 5 Dentist, Yazd, Iran

**Keywords:** Panoramic radiographs, Nerve, Inferior alveolar, Mandibular nerve

## Abstract

**Statement of the Problem:**

The inferior alveolar canal (IAC) is a bony canal that starts from mandibular foramen at the inner surface of the mandibular ramus and extends along the ramus and body of mandibular bone in forward and downward directions to the mental foramen. Inside the mandibular canal, there are lower alveolar artery and a nerve with the same name. Understanding the anatomical details of the lower alveolar canal, including position, pathway and morphology to prevent complications in surgical procedures in the lower jaw such as mandibular impacted molar surgeries, mandibular nerve block injection, or even root canal treatment of mandibular teeth is important.

**Purpose:**

The purpose of this study was to investigate the course and direction of IAC in mandibular bone and its relation to anatomical factors such as gonial angle and location of entrance of IAC in the mandibular ramus.

**Materials and Method:**

This cross-sectional study evaluated a sample of 280 digital panoramic images. All samples were Iranian. The pattern and direction of the IACs were recorded according to age and gender and the relation of these patterns to the gonial angle of mandible and the entry point of the IAC in mandibular ramus were evaluated.

**Results:**

The results showed that the course of canal, the entrance point of the canal and the gonial angle were the same between different age groups and between two genders. There was no significant relation between the course of canal and the two anatomical variables mentioned (*p*> 0.05).

**Conclusion:**

Considering the increasing frequency of implant surgeries and presence of different courses of the mandibular canal and concerning the important complications such as paresthesia caused by damage to the mandibular nerve, panoramic radiography is necessary before any surgery in this area to avoid unwanted injuries to the neurovascular system if other advanced modalities are not available.

## Introduction

The inferior alveolar canal (IAC) is a bony canal that starts from the mandibular foramen at the medial surface of the mandibular ramus and extends along the mandibular ramus from mandibular foramen in forward and downward directions to the mental foramen. Inside the mandibular canal, the inferior alveolar artery and nerve are present. The inferior alveolar artery provides blood supply to the mandibular teeth and related structures [ 1 ]. Understanding anatomical details of the IAC including position, course, and morphology is useful in mandibular impacted molar surgery, mandibular nerve block injection, mandibular bone resection, mandibular teeth root canal treatment, and other mandibular surgical procedures [ 2 - 3 ].

 According to Liu *et al*. [ 4 ] study, the course of IAC can be divided into four groups: (1) Linear Curve, (2) Spoon Curve, (3) Oval Curve, and (4) Turn Curve. If vital structures such as inferior alveolar nerve and mental foramen are not accurately identified, many disorders such as altered sense of mandibular tissues, mandibular anesthesia, stinging, and pain in the mandible usually occur after surgery. In addition, damage to the related blood vessels, such as inferior alveolar artery or lingual artery, which may have a high potential for bleeding, can be a result of failure to identify the anatomical location of these structures. Therefore, detection of the position and configuration of the IAC and related anatomical structures is crucial for reducing such damage to this canal [ 5 ]. In some radiographs, the IAC has a cortical border, but in other radiographs, especially in patients with osteoporosis, it can be confused with bone marrow [ 6 ]. In addition, anatomical differences of the IAC may be a factor for failure of inferior alveolar nerve block injection [ 7 ]. Although the morphology and position of the canal vary in different ethnic groups and in different jaw types, these changes are often overlooked and cause problems in dental treatment. A detailed understanding of the factors affecting the anatomical changes in morphology of the canal can minimize this problem [ 8 ]. The purpose of this study was to investigate the course and direction of IAC in mandibular bone and its relation to anatomical factors such as gonial angle and location of entrance of IAC in the mandibular ramus.

## Materials and Method

In this cross-sectional study, digital panoramic images taken from dental patients (2015-2017) were obtained from the archives of Oral and Maxillofacial Radiology Department, Faculty of Dentistry, Yazd, Iran. A total of 280 panoramic images were selected by random sampling. All samples have been selected from Iranian population and composed of men and women aged 18-60 years. The type of IAC course was diagnosed by visual detection and comparison with references. Panoramic images had been taken by Planmeca-Promax (Helsinki, Finland) with the same conditions (80 kvp, 12mA, 18 s). Planmeca Romexis Viewer 451R (Helsinki, Finland) software was used to evaluate the course of the inferior alveolar canal, gonial angle, and IAC insertion. These factors were evaluated in left side of each patient.

**Figure 1:**
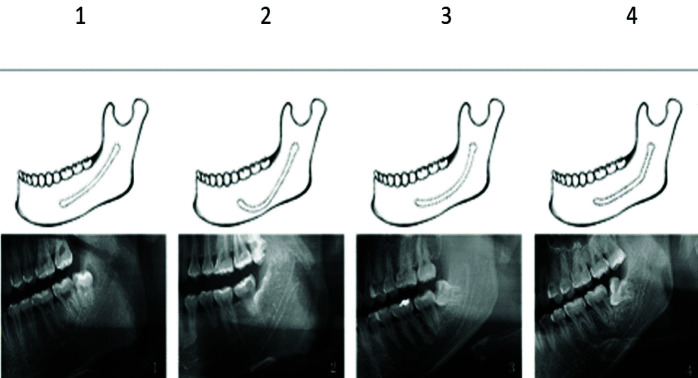
Types of canals

According to the study of Liu *et al*. [ 4 ], the course of IAC was classified into four categories based on its appearance on panoramic radiography defined as Type (1) linear curve (right) : a canal that is in contact or in close contact or maximum at a distance of 2 mm to the apex of the first mandibular molars; Type (2) spoon-shaped curve: canal that is in contact or in close contact or maximum at a distance of 2 mm to the inferior mandibular cortex; Type (3)oval curve(curved): the status between modes 1 and 2 (position intermediate); and Type (4) turning curve( angled) (Figure 1).

Measurements were performed using Romexis Viewer software on the Panoramic Digital Imaging Processor on computer at Yazd Dentistry School by selecting the ruler menu and the angle measurement menu and drawing lines in the desired areas.

### Gonial Angle Measurement

**Figure 2:**
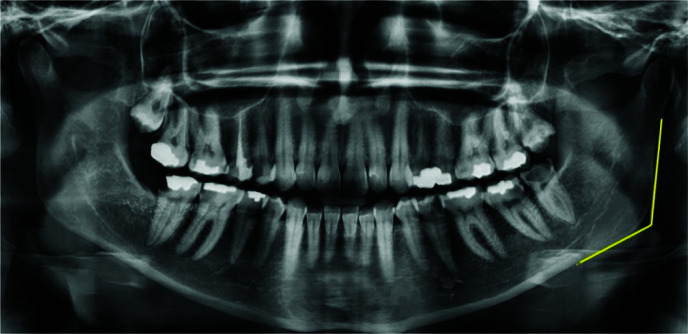
Gonial angle

The gonial angle was measured on a degree scale and recorded in a checklist. Gonial angle was measured by measuring the angle resulting from the collision of two tangent lines on the mandibular inferior border and posterior ramus border [ 9 ] (Figure 2).

### IAC entry point

**Figure 3:**
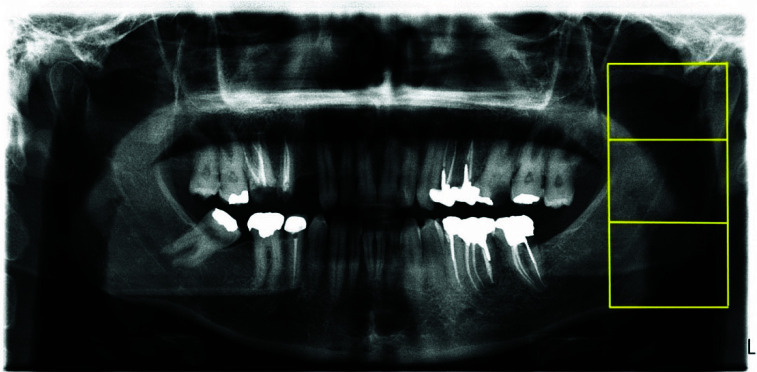
Inferior alveolar canal origin location

To check the canal entry point, the distance from the condylar most upper point to the mandibular angle was categorized into three equal parts as the upper, middle, and lower parts. Then the canal entry location was classified according to the specific region it was in, and recorded in the checklist [ 10 ] (Figure 3). 

After observing all samples, data on patient gender and type of canal course were recorded in a checklist. Data were analyzed by SPSS software version 20; Chi-square, ANOVA and t test were used for data analysis.

## Results

This study was performed on 280 digital panoramic images from the Department of Radiology, Shahid Sadoughi Dental School, Yazd. The mean age of the samples was 35.58±10.66 with a range of changes from 18 to 60 years. 155 samples were female (55.4%) and 125 samples were male (44.6%).

The results showed that the most common type of canal was curved type (69.28%) and the less common was spoon type (6.78%). In this regard, in all age groups, the most common type of canal was curved type. These differences were tested by the Chi-square test, with *p*= 0.113, which is not significant, which means different types of lower alveolar canal in different age groups were identical (Table 1). Also in evaluation of the frequency distribution of lower alveolar canal types according to gender, the same results were obtained (*p*= 0.113) so that different types of lower alveolar canal in two genders were identical. The most common type of canal in men and women was the curved type and the less common type was spoon and right type, respectively (Table 2). 

**Table 1:** Frequency distribution of the alveolar canal type according to age

Type of canal	Right n (%)	Angled n (%)	Curved n (%)	Spoon n (%)	Total n (%)
Age Group					
20-29	4 (4.2)	14 (14.8)	69 (73)	7 (7.4)	94 (33.5)
30-39	10 (9.4)	19 (17.9)	72 (67.9)	5 (4.7)	106 (37.8)
40-49	7 (14)	6 (12)	33 (66)	4 (8)	50 (17.8)
50 and above	5 (16.6)	2 (6.6)	20 (66.6)	3 (10)	30 (10.7)
Total n	26	41	194	19	280

*p*= 0.113

**Table 2:** Frequency distribution of lower alveolar canal types according to gender

Type of Canal	Gender		Total
	Female	Male	
Right	14(9)	12(9.6)	26(9.3)
Angled	27(17.4)	14(11.2)	41(14.6)
Curved	108(69.7)	86(68.8)	194(69.3)
Spoon	6(3.9)	13(10.4)	19(6.8)
Total n	155	125	280

*p*= 0.113

The maximum average of gonial angle belonged to the age group of 30-39, the lowest mean belonged to 50 years and above, which after analyzing by ANOVA test (*p*= 0.134) we concluded that the mean gonial angle was the same in different age groups. In addition, same result was yielded for two genders using t test (*p*= 0.67).

Table 3 shows the average gonial angle according to canal type .The results of ANOVA test (*p*= 1.0) showed that the frequency of different types of canals were the same at different gonial angles. In addition, the results of ANOVA test showed that there was no significant relationship between the location of canal origin and canal type (*p*= 0.23).

**Table 3:** Average gonial angle and canal origin frequency distribution according to canal type

Type of canal	Frequency	Average Gonial Angle (Degree)	Canal Origin	
			Upper	Middle
Right	26	134.8	2	24
Angled	41	121	0	41
Curved	194	121.3	5	189
Spoon	19	119.5	1	18

*p*> 0.05

The results showed in %97.15 of cases, the middle third area of ramus was the entrance point of canal and the entrance point of canal in the lower third area was not observed in any of the images. No significant difference was observed in men and women considering the entrance point of the canal. This correlation was analyzed by Chi-Square test and was not significant with *p*= 0.76, meaning that the location of entrance of canal in different genders were the same. Moreover, this was also true about different age groups (*p*=0.76); the locations of canal entrance in different age groups were the same.

## Discussion

The location and the course of the IAC are important factors in surgical procedures in posterior part of mandible [ 11 - 12 ]. Therefore, prior to any procedure in this area, the frequency of anatomical variations of the IAC course should be considered [ 13 ].

Radiological diagnosis of a disease requires accurate knowledge about the radiographic normal structures and its variations [ 14 ]. In our study, the mean gonial angle in the images was 122.37, which was not significantly different between men and women. Oettle *et al*. [ 15 ] stated in their study that there was no significant difference between the two groups of gender in gonial angles, which is in line with the results of our study.

However, Gungor *et al*. [ 16 ] stated that the difference in gonial angles of the male and female was statistically significant, so that the average gonial angle in women was greater than that of men. This difference with the results of our study could be attributed to the different races or the number of samples of two studies. Knowing the position of IAC in different points of the path and the knowledge of canal diversion points help dentists identify high-risk areas. This is especially important in procedures such as surgery, endodontics, endodontic surgeries, dental implants, and so on [ 17 ]. The results of our study showed that, in terms of the entrance of the canal, the middle third area of ramus had the highest incidence (97.1%) for entrance of ​​the canal. The study of Mbajiorgu [ 18 ] stated that the entrance of the canal in 94% of the panoramic images was about 3 mm higher than the middle third part of ramus. In this regard, the results of our study are consistent with the outcomes of their study. However, Kilic *et al*. [ 19 ] reported that 83% of the entrance points of canals were 10.52 mm higher than the inferior margin of mandible, which is not consistent with the results of the present study. This difference can be related to the different methods used in two studies. 

In the current study, we applied the Liu’s classification [ 4 ] to categorize the course of IAC. Based on the results of this study, the most common course was curved type (oval type) (69.3%) which is in line with the results of Liu *et al*. (48.5%); while the small difference in numbers could be due to different number of samples in two studies. 

The anatomical position of the IAC canal appears to differ with age [ 20 - 21 ]. In this study, we evaluated the IAC course in four age groups. Oval type (curved) pattern was the most prevalent pattern in all age groups. In Mirbeigi *et al*.’s study [ 22 ], which was done on CBCT images, straight (linear) and progressive descending (spoon type) patterns were the most prevalent patterns in the 20–29 age group. In the 30–44 and 45–59 age groups, the most prevalent category was recorded as straight and catenary (curved type), respectively. There was not any statistically significant difference between the three age groups in their study, which is in line with the results of the present study. 

Various factors such as gender, age, and some anatomic factors could affect the IAC course. Although none of these factors had any significant effect on the results of this study, the methods of examination of IAC could affect the obtained results. Therefore, further studies using other methods such as CBCT with consideration of these interfering factors should be designed in a larger population.

## Conclusion

Due to increasing number of implant surgeries and different courses of the mandibular canal and important complications such as alterations in sense caused by damage to the inferior alveolar nerve, panoramic radiographs are required before any surgery to prevent complications when CBCT and other advanced imaging modalities are not available.
